# From wearable sensor data to digital biomarker development: ten lessons learned and a framework proposal

**DOI:** 10.1038/s41746-024-01151-3

**Published:** 2024-06-18

**Authors:** Paola Daniore, Vasileios Nittas, Christina Haag, Jürgen Bernard, Roman Gonzenbach, Viktor von Wyl

**Affiliations:** 1 Institute for Implementation Science in Health Care, University of Zurich, Zurich, Switzerland; 2 Digital Society Initiative, University of Zurich, Zurich, Switzerland; 3 Department of Behavioral and Social Sciences, Brown University, Providence, USA; 4 Epidemiology, Biostatistics and Prevention Institute, University of Zurich, Zurich, Switzerland; 5 Department of Computer Science, University of Zurich, Zurich, Switzerland; 6 Valens Rehabilitation Centre, Valens, Switzerland; 7 Swiss School of Public Health (SSPH+), Zurich, Switzerland

**Keywords:** Diagnostic markers, Clinical trials, Diagnostic markers

## Abstract

Wearable sensor technologies are becoming increasingly relevant in health research, particularly in the context of chronic disease management. They generate real-time health data that can be translated into digital biomarkers, which can provide insights into our health and well-being. Scientific methods to collect, interpret, analyze, and translate health data from wearables to digital biomarkers vary, and systematic approaches to guide these processes are currently lacking. This paper is based on an observational, longitudinal cohort study, BarKA-MS, which collected wearable sensor data on the physical rehabilitation of people living with multiple sclerosis (MS). Based on our experience with BarKA-MS, we provide and discuss ten lessons we learned in relation to digital biomarker development across key study phases. We then summarize these lessons into a guiding framework (DACIA) that aims to informs the use of wearable sensor data for digital biomarker development and chronic disease management for future research and teaching.

## Introduction

The increasing popularity of ubiquitous mobile technologies, such as wearables, has the potential to transform chronic disease management^1–3^. The broad adoption of wearables, particularly commercial activity trackers, is driven by their affordability, user-friendliness, and overall high accuracy^4^. The rising amount of research on chronic diseases that involves wearables highlights this trend^5–7^. Wearables are equipped with sensors that generate health-related data in real-time, creating opportunities for personalized care^8^. The clinical relevance of this data ultimately depends on their translation into digital biomarkers^9,10^. This process generally requires the definition of *normal* ranges, which is either informed by external benchmarks (e.g., 10,000 daily steps) or intra-individual norms (e.g., individual average step counts during the week) that can be further validated with patient-reported data (e.g., surveys)^11–13^. However, most wearables have fixed measurement capabilities (e.g., physical activity and heart rate), which currently limit their translation to digital biomarkers.

For the potential of digital biomarkers to be achieved, aligning wearable capabilities and study design with recommended practices for meaningful clinical measures is essential^14^. The Food and Drug Agency (FDA) guidance document on the use of digital health technologies for remote data acquisition in clinical investigations proposes a multi-step approach towards digital biomarker development, in which the validation and verification steps take central roles^15^. Along similar lines, the framework by the Digital Medicine Society on best practices for evaluating monitoring technologies for use in clinical trials emphasizes verification, analytical validation, and clinical validation (V3) as central steps^16,17^. While these documents provide useful high-level guidance, they offer limited support for the development of digital, wearable-based biomarkers. Furthermore, in current guidance there is an absence of study design and conduct elements that involve all stakeholders in an iterative approach and focus on the implementation of digital biomarkers in practice. Consequently, researchers and health professionals often rely on limited guidance for the use of wearable data in clinical practice and chronic disease management^18,19^.

Digital biomarkers may significantly improve the management of complex chronic conditions, such as multiple sclerosis (MS). MS is a serious neurodegenerative health condition that is characterized by both extensive and highly variable physical and mental symptoms. More than 15,000 people are currently living with MS in Switzerland alone^20^. Optimizing and tailoring treatment options has been limited by a still unexplained heterogeneity in symptom patterns and disease course. For this reason, MS is often referred to as the ‘disease with 1000 faces’^21^. In this paper, we briefly introduce the BarKA-MS study program (section “Introduction”), which collected sensor data from wearables on the physical rehabilitation of people living with MS (PwMS), and summarize ten important lessons learned (section “The BarKA-MS study program”) across key study phases related to methods aimed at guiding the development of digital biomarkers^22^. We then present the DACIA framework (section “Lessons learned from BarKA-MS”) as a crosscut between the ten lessons and five crucial steps of digital biomarker development, which has been applied twice in the course “Digital Health in Practice” for medical students at the University of Zurich. Finally, we discuss the DACIA framework in the context of existing guidance and highlight its relevance. Our work aims to inform (1) future research on the development wearable-based digital biomarkers for chronic disease management, as well as (2) teaching curricula, through the application of our framework^10,11^.

## The BarKA-MS study program

BarKA-MS is a semi-remote observational, longitudinal cohort pilot study program that explored the physical activity rehabilitation of PwMS, which informed several independent analyses as part of the program^18,23–26^. The methods and results of BarKA-MS are published elsewhere^22,24–26^. The study was planned in collaboration between the researchers, clinical staff, as well as experts in human-centered and interactive visual data analytics (IVDA). During study design, clinicians and researchers defined relevant clinical measures for potential future use in a rehabilitation clinic. Study nurses from the clinical staff were consulted to identify feasible data collection methods, drawing on their experiences with PwMS and their understanding of patient needs. Data collection was planned with the Fitabase activity tracker database^27^ to enable the statistical analysts and IVDA experts to effectively translate wearable sensor data to digital biomarkers.

BarKA-MS was divided in two phases. First, the physical activity of participants was measured during their inpatient rehabilitation stay at the Valens Rehabilitation Centre in Switzerland, which for most patients lasted between two to three weeks. Second, their physical activity was measured upon return to their homes. Participants were asked to wear the Fitbit Inspire HR during the entire duration of the study^28^ and an additional research-grade wearable sensor, the Actigraph GTX, during their last week of rehabilitation and the first week back home^25^. Participants were followed up for up to eight weeks i.e., two to four weeks in the first phase and four weeks in the second phase. Technical and motivational support was provided throughout the study. The study protocol obtained ethical approval from the Zurich cantonal ethics commission (BASEC-no. 2020–02350). All participants provided written informed consent.

Participant demographics of BarKA-MS are available in Supplementary Table 1. At baseline, most participants were female, had a median age of 46, had MS for a median of 11 years and were either working part-time or were unemployed. These characteristics align with the typical demographics observed in MS populations with a more progressed disease state^29–31^. A follow-up study^23^ involving participants with different characteristics and chronic illnesses, such as cardiovascular diseases, revealed conclusions consistent with the main BarKA-MS analyses, suggesting that the findings discussed in this lessons learned paper may be applicable to other chronic disease populations.

Relevant wearable sensor data was collected longitudinally and included heart rate, step count, sleep indicators, physical activity intensity (time spent in light, moderate, or vigorous physical activity), and sedentary time. These measurements were available at the minute, hourly, and daily granularity levels. To provide additional context to the physical activity measures from the wearable sensors, we collected self-reported data using the following instruments: (1) the 18-item Barriers to Health Promoting Activities for Disabled Persons Scale^32^ to assess perceived barriers to physical activity, (2) the 12-item MS Walking Scale-12^33^ to assess the walking ability of the participants and (3) the Fatigue Scale for Motor and Cognitive Functions^34^ to assess MS-related cognitive and motor fatigue. The study achieved a weekly survey completion of 96%, as well as 99% and 97% valid Fitbit wear days at the rehabilitation clinic and in the home setting, respectively.

## Lessons learned from BarKA-MS

In the following sections, we present our insights (lessons learned) from designing and implementing BarKA-MS, as well several independent analyses of sensor measurements and patient reported outcomes^18,24–26^, and a follow-up study that was modeled after BarKA-MS^23^ that examined the implementation of a physical activity post-rehabilitation program from the perspectives of patients and healthcare professionals. We specifically selected insights that are relevant to the use of wearable sensor data for digital biomarker development. All our lessons learned were discussed and co-formulated with healthcare professionals, clinical staff and researchers involved in BarKA-MS, and categorized in four key study phases, including: (1) early study design, (2) study execution, (3) data analysis, and (4) data interpretation.

### Early study design

For BarKA-MS, we chose to use the Fitbit Inspire HR commercial wearable after an assessment against other devices due its low cost, ease of use and ability to collect relevant data with Fitabase^27^, a secure third-party data collection tool that enables remote monitoring of data quality and completeness checks. By contrast, the Actigraph accelerometer was not chosen as the primary wearable device for data collection due to its higher costs, lower participant preference from discomfort of wearing it around the hip, and increased complexity due to limited storage capacity and the requirement to actively download data with a cable. These initial decisions were taken during the protocol writing phase and in agreement with healthcare professionals and clinical staff. Central to these decisions was also designing the study to protect the privacy of the participants, by ensuring the safe collection and use of data. In particular, only non-identifiable user accounts were used for wearable devices and potentially sensitive features of the devices, such as location tracking or data sharing via social media, were disabled. These decisions led to the following lessons.

#### Lesson 1: Aligning study goals and technology

The choice of measurement tools should be guided by the research question and the study outcomes of interest. In our case, the primary outcome was daily-life physical activity, a proximal outcome that was directly derived from the Fitbit Inspire HR. To decide whether a wearable is the most suitable option, it is key to fully understand the functions, but most importantly the potential limitations of devices. Understanding the limitations reduces the risk of unreliable measurements. A relevant example comes from one of our previous unpublished sub-analyses of BarKA-MS, which examined correlations of self-reported fatigue (using the Multiple Sclerosis Impact Scale-29 score^35^) and sensor measurements, including sleep length and daily-life physical activity. Our findings revealed weak associations, which were likely due to the wearable’s indirect measurement of distances^26^. Having missed this limitation would have likely led to incorrect measurements.

#### Lesson 2: Aligning measurement and outcome assessment timeframes

A second lesson learned during the early design phases of BarKA-MS is the importance of required timeframes, or the time needed until relevant study outcomes can be fully measured. Chronic diseases, such as MS, progress over years or decades. Recent digital health studies on chronic diseases have reported monitoring periods of up to 12 months^2^. However, the optimal timeframe to detect a change of interest depends on the study question. In the case of BarKA-MS, we detected clinically relevant changes in self-reported measures related to barriers to physical activity for severe fatigue scores in 8 out of the 38 participants, and a median improvement of 16.7 points in the MS Walking Scale-12 after an 8-week follow-up^24,26^. By contrast, health behaviors, such as daily-life physical activity, fluctuate on much smaller time scales, such as days, weeks, or months. Nevertheless, our experiences with BarKA-MS and a follow-up study^23^ suggest that even timeframes of 4 to 12 weeks require significant efforts to keep participants engaged. Being aware of the expected efforts during the study, the availability of resources, and the characteristics of the study population, such as their age, level of disability and educational level, will ultimately determine whether (a) the use of wearables is scientifically meaningful, and (b) what duration periods will likely be needed^24^. Commercial wearables are well-geared towards measuring health behavior changes on weekly or monthly time scales, while also supporting longer study durations due to their ease of use and wear comfort. Not defining timeframes correctly and early enough risks delays and waste of resources.

#### Lesson 3: Defining the role of wearables

**Fig. 1:**
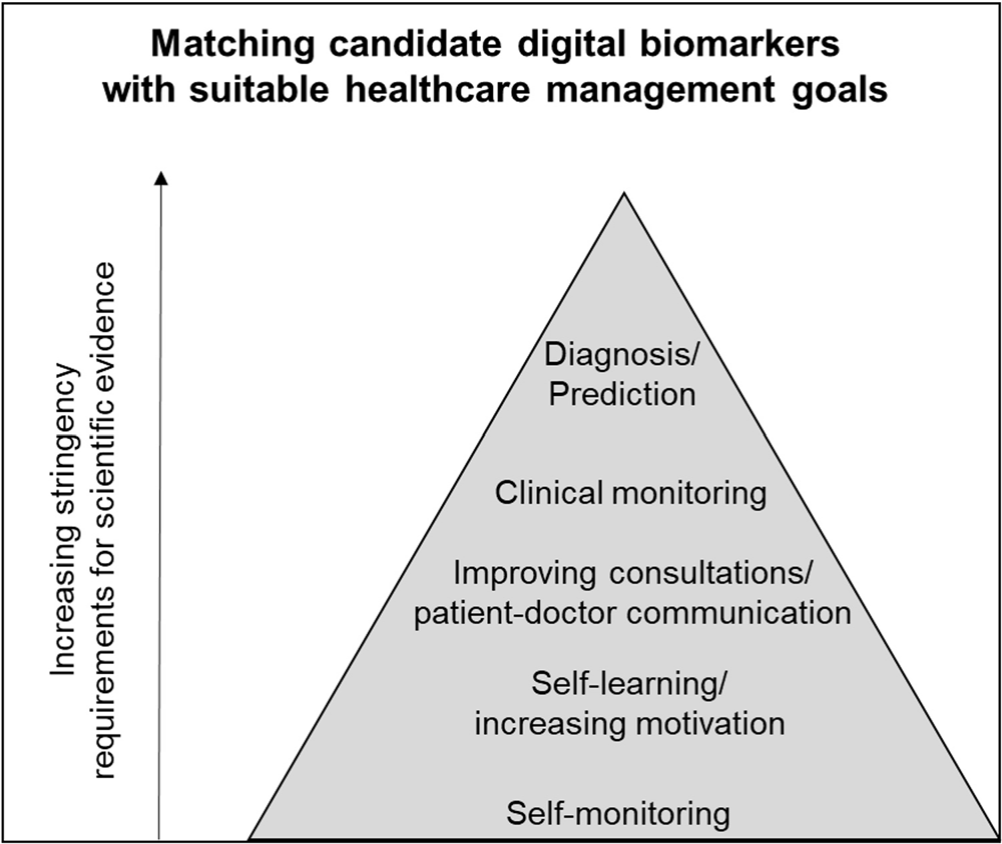
Goal versus effort pyramid to inform the role of wearable sensors in achieving research goals.

Wearables can take different roles and thus, support different goals in chronic disease management. In our discussions with healthcare professionals involved in BarKA-MS, we identified the need for clarity regarding the role of wearables in digital biomarker studies. Two central questions emerged: “how can sensor data improve patient health?”, and “who should take action to achieve health benefits?”. These questions led to the development of our “goal pyramid” (Fig. 1), which outlines various healthcare goals that wearable data can support. These goals range from low-effort (bottom of the pyramid), to high-effort, yet clinically more informative, goals (top of the pyramid). For example, prediction studies might require longer follow-up times, larger sample sizes, and additional data for prediction model validation. Overall, the “goal pyramid” is a useful tool to facilitate discussions with healthcare professionals about study designs and for clarifying technology’s role in achieving health outcomes, along with the associated efforts.

### Study execution

Not all study execution challenges can be anticipated during the design phase. For example, BarKA-MS offered comprehensive participant support, which resulted in high study compliance. However, we recognize that this approach is likely not an option for studies with larger samples. Overall, our experiences, based also on feedback from clinical staff, point to a trade-off between collecting high-quality and near-complete data while optimizing participant burden and maintaining high compliance. The following two lessons reflect our experiences during study execution.

#### Lesson 4: Combining passive monitoring with actively collected data

BarKA-MS taught us that the combination of wearable sensor data with other data types (e.g., clinical, physiological, or patent-reported data) may enhance the accuracy of digital biomarker development. Rationales for collecting additional data types may include sensor validation, multivariable predictions of health outcomes, or stratification through subgroup analyses. In BarKA-MS, we deliberately used commercial wearables not specifically designed for use by PwMS. To enhance and contextualize the rather generic wearable sensor data, we collected patient-reported symptoms, frequency of physical activity, and its associated barriers, along with free-text feedback on wearable use and acceptability. In BarKA-MS, assessing this combination of passively and actively collected data was a crucial first step in exploring possible digital biomarkers of barriers to physical activity in the context of shifts in fatigue and mobility^26^. However, previous examples have also demonstrated that active data collection, such as through surveys, carries a risk of drop-outs or non-compliance^36^ that may be higher than in studies with only passive data collection (e.g., wearables). Although a recent scoping review^4^ was unable to identify clear associations of participant burden due to active data collection, this aspect should be carefully monitored and possibly adjusted during the study.

#### Lesson 5: Maintaining and supporting participant compliance

Data completeness and participant compliance are particularly relevant, especially for studies that are conducted remotely. A key initial consideration for digital health studies is ensuring that participants are representative of the study’s target population, including relevant underrepresented groups^37^. This may require targeted recruiting efforts, as well as possible contextual and cultural adaptations of the study design^38^. In BarKA-MS and a follow-up study^23^, efforts were taken to enhance the diversity of the study population in terms of age and gender by providing participant onboarding and technical support during follow-up. Participants also provided weekly feedback about their experience with and usability of the Fitbit. Problems were either addressed by the clinical staff at the rehabilitation clinic or the two involved researchers. For example, when participants encountered technical issues with their Fitbit, researchers promptly scheduled phone calls to resolve the problems^23,24^. As shown by an internal assessment of support logs, these measures helped retain older or more impaired study participants with higher MS symptom burden^24^. BarKA-MS achieved high study compliance but also required considerable efforts to actively monitor data collection (e.g., frequent personal reminders from the researchers). Missing data and dropouts are also inevitable. Declining participant motivation or health, inconvenient timing, or burdensome data collection can all contribute to low compliance and missing data. In BarKA-MS, declining health often demotivated participants who preferred not to receive physical activity reminders, as these highlighted their physical limitations. This further illustrates that challenges may emerge and even multiply over longer observation periods, underscoring the need for continuous participant support.

### Data analysis

For BarKA-MS, we focused the data analysis on: (1) time series assessments of wearable sensor data for recurring patterns within/between PwMS, and (2) descriptive analyses to explore physical activity barriers for PwMS. To better visualize and assess these results, we conducted an unpublished sub-study in collaboration with experts in IVDA. These were then discussed with IVDA experts and healthcare professionals to better understand the present data quality and analytical challenges, and contribute to the formulation of new hypotheses. The following lessons reflect these experiences.

#### Lesson 6: Defining appropriate data aggregation level

Wearable sensors collect data at different time scales. For example, step count, time spent in active physical activity, and heart rate are available at the minute level, while resting heart rate, which is measured at nighttime, is only available as a single daily value. Finding the most appropriate temporal aggregation level depends on the expected timeframe needed to observe an effect in the outcome of interest (lesson 2), as well as mitigating redundancy and low data resolution^39^, or ensuring that outcome measures comply with those relevant in clinical settings^40^. In BarKA-MS, we collaborated with healthcare professionals to create interactive visualizations from the study’s sensor data. These experiences highlighted that daily aggregations were meaningful for most parameters to develop informative composite measures, but longer-term assessments might benefit from weekly or even monthly data aggregations, with the option to switch between aggregation levels. Further considerations include whether data aggregation can help with managing high volumes of data. Data aggregation can help with reducing information overload, which can help healthcare professionals and patients understand the data and its signals more easily. In BarKA-MS, we followed a user-centered design methodology to co-design sensor data visualizations together with healthcare professionals, to facilitate informed decision-making based on meaningful data signals. The resulting data visualizations also revealed useful for guiding researchers in analyzing BarKA-MS data.

#### Lesson 7: Contextualizing sensor measurements

In BarKA-MS, the main challenge of developing digital biomarkers was the contextualization of our data. A common issue was distinguishing between patterns in physical activity due to exercise or unrelated activities, such as knitting or playing the piano. This was highlighted in a BarKA-MS analysis that revealed weak correlations between different sensor measurements in a real-world setting^25^, echoing similar reported difficulties in the scientific literature^41–43^. Another challenge involved connecting irregular patterns of activity or inactivity with individual or group-level factors that influence motivation. For example, among PwMS there is a high prevalence of fatigue (affecting over 70% of PwMS^44^), which may demotivate them from exercising, as observed in a BarKA-MS analysis revealing a positive correlation between levels of fatigue and barriers to physical activity^26^. Individual-level visualization of the data with healthcare professionals as part of BarKA-MS highlighted the need for contextual information related or unrelated to sensor measurements to help identify patterns of interest for individual participants^45^. For example, visualizations of physical activity and sleep data from BarKA-MS suggested cyclical within-person patterns, such as higher physical activity on weekends. In BarKA-MS, we also used weather condition data to assess whether deviations in activities could be contextualized to other, external influencing factors. Knowledge about the temporal occurrence of such factors may overall help to better interpret sensor measurement data.

#### Lesson 8: Discerning signal from noise

Filtering out “noise”, or signals in the data collection that are of low value and are not indicative of the presence of an actual signal^46^, within sensor data is a key, yet challenging task. Building on lesson 7, contextual data, such as weather patterns, can help distinguish between trivial explanations for patterns, or nuisance parameters, and the actual patterns of interest to the study^47^. For example, by applying interactive visualizations to our BarKA-MS data we observed differences in step counts or sleep patterns between weekdays or weekends. In some individuals, healthcare professionals also noticed distinct within-day patterns, such as reduced activity in afternoons, which they identified as possible signs of fatigue, a common symptom in PwMS. Another approach is to build a time series model that includes these noise parameters to predict expected sensor measurements. This de-noising approach involves gathering and analyzing data from nuisance variables that introduce noise, such as daily routines, weather and calendar data, alongside sensor measurements. The inclusion of such nuisance variables, if they are indeed associated with the outcome, has the potential to decrease noise. Ideally, the identification of variables required for “de-noising” should be considered at the study planning stage.

### Data interpretation

The data interpretation phase is linked with the analysis phase, however, focuses more on the contextual interpretation of results. For BarKA-MS, visual data analytics and discussions with healthcare professionals played a key role. We derived the following two lessons.

#### Lesson 9: Choosing internal and external benchmarks

Digital biomarkers should ideally be characterized by clear norm ranges. However, it is difficult to develop universal norms, as observed with healthy individuals occasionally having laboratory values outside the norm, or the other way around. Data interpretation is further challenged by possible systematic measurement inaccuracies, such as those from Light Emission Diode-based wearable devices that may be less accurate for people of color^42,48^, or datasets omitting underrepresented groups^49^, which can contribute to biased benchmarks. Considering these challenges, digital biomarker studies should focus on inter-individual changes rather than absolute benchmarks^50,51^. In BarKA-MS, physical activity level digital biomarkers were informed by internal and external benchmarks. Internal benchmarks were derived to assess if individual PwMS exhibited certain patterns that occurred more frequently than expected, considering a normal distribution. External benchmarks were obtained directly from the wearables, using calculated measures of e.g., physical activity intensity, such as the amount of time spent in light, moderate, or vigorous physical activity^25^. These measures served as digital biomarkers for low or high levels of physical activity. For such metrics in chronic disease populations, such as MS, personal contexts play an important role. This underlines the need for studies on chronic disease populations to assess changes in intra-individual norms and, ideally, health status assessments from clinicians to develop meaningful digital biomarkers.

#### Lesson 10: Deriving clear actions

For digital biomarkers to be of clinical value, they should be linked to an action plan. Such an action plan may include defining the rules that confirm digital biomarker deviations (e.g., outside-norm signals in two subsequent weeks), monitoring frequently, and adjusting intervention delivery (e.g., motivational phone call to participant). Building on lesson 3, such action plans should be aligned with the overall goal of the study and the role of wearables, as illustrated by the “goal pyramid” (Fig. 1). For BarKA-MS, the interactive data visualizations and discussions with healthcare professionals revealed important preconditions for reacting to digital biomarker changes. For example, healthcare professionals stated that such processes should be compatible with existing workflows to avoid additional burden to clinical staff and healthcare professionals themselves, or that technical support for both patients and clinical staff should be made available^23^. A follow-up study explored these topics using the normalization process theory framework, focusing on how healthcare professionals and patients can collaborate effectively in remote activity tracking for rehabilitation aftercare^23^.

## The DACIA framework to inform planning of wearable sensor data use in healthcare research, management and teaching

**Fig. 2:**
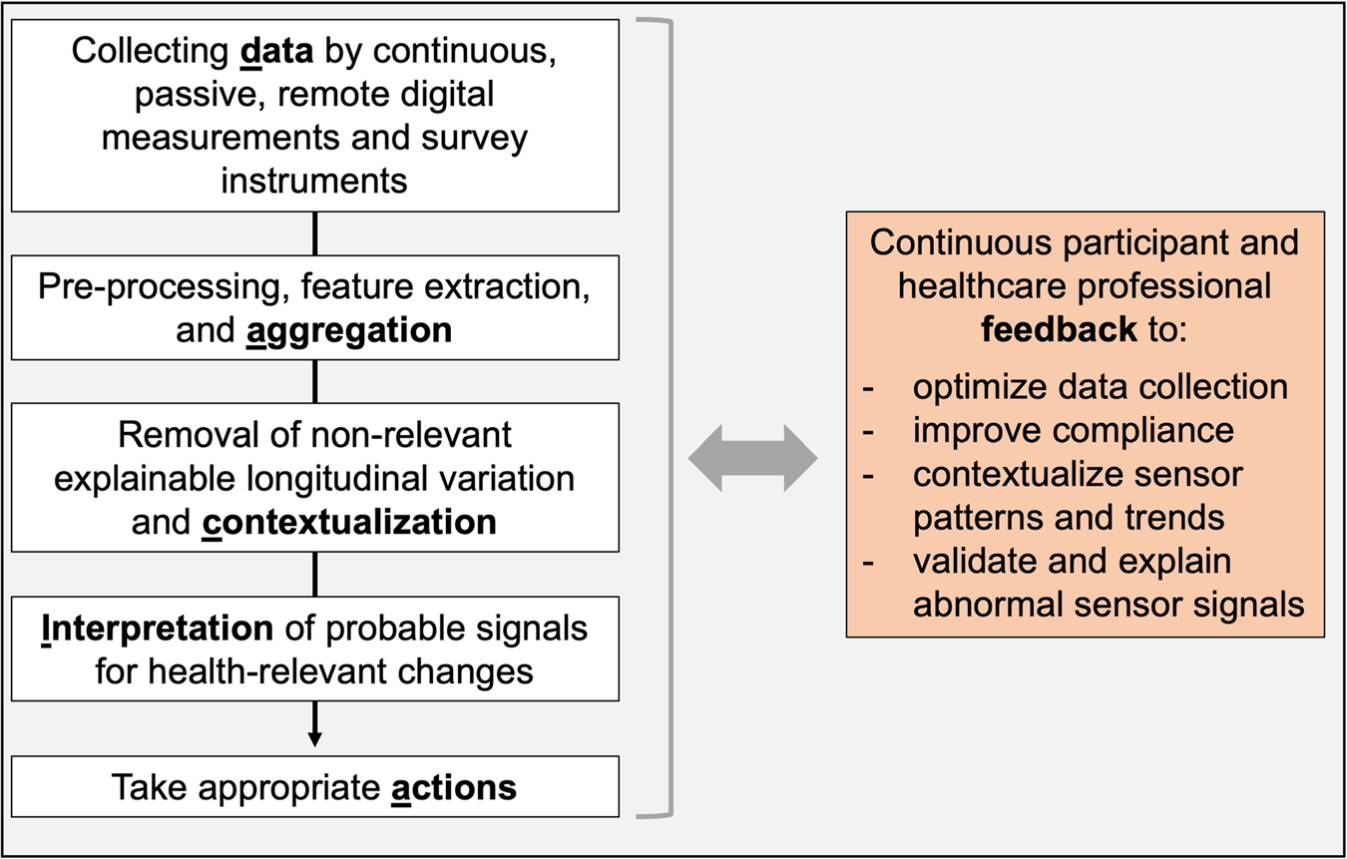
DACIA framework constructs and feedback loops.

Drawing on identified patterns and themes from the ten lessons from BarKA-MS, observations from a follow-up study^23^, and feedback received when used in the course “Digital Health in Practice” for medical students at the University of Zurich, we developed the *DACIA* framework. This framework is based on the notion that digital biomarker development is informed by: (1) data, (2) aggregation, (3) contextualization, (4) interpretation, and (5) actions (Fig. 2). These constructs aim to guide future early-stage research on wearable sensor-based digital biomarker development and are scalable to larger studies. The DACIA framework also serves as an interactive teaching tool for medical students to plan and execute a hands-on wearable sensor data collection and analysis for a mock digital health intervention.

In this section, we present the five DACIA constructs along with examples for guiding questions to inform study planning (Table 1), which can also be used to support teaching. We then present data loops among the DACIA constructs, depicted by the orange box, to illustrate the iterative and flexible aspects of digital biomarker development. To provide further context on DACIA’s applicability to a study, we apply the constructs of the framework to BarKA-MS (Supplementary Table 2).

**Table 1:** Description of DACIA framework constructs and relevant guiding questions

Construct (related lessons)	Guiding questions
Data collection (lessons 1–5): The *data collection* step consists of selecting the most suitable wearable sensor measurements and time frameworks for digital biomarker development. Additional relevant clinical or patient-reported data should be considered for validation purposes.	Which wearable sensor data are best suited to monitor relevant health and behavior change outcomes? What time frame will be required? Which other data types can meaningfully complement the wearable sensor data for higher digital biomarker accuracy? What could contribute to missing data?
Aggregation (lessons 3–6): The *aggregation* step includes the selection of the most suitable temporal analysis level, combination of measurements, as well as further feature extractions and transformations (e.g., by using multivariable methods).	Which temporal granularity (e.g., second, minute, hour, day) is most suitable? Does the wearable provide this aggregation level? For higher-level granularity: what is the minimal wearable wear-time?
Contextualization (lessons 2, 6, and 7): The *contextualization* step involves combining wearable sensor data with details such as measurement timing (e.g., seasonality), patient traits, population norms, and normal intra-individual ranges (e.g., average weekday step counts, sleep duration).	Do the main signals depend on measurement timing (weekday, season) or specific participant characteristics (gender, age, body mass index (BMI))?
Interpretation (lessons 7–9): The *interpretation* step includes the development of rules to discern signals from noise. This step is aided by contextual and intra-individual baseline data, as well as feedback from healthcare professionals and the wearable users themselves. For example, presenting patients with their data can validate signal detection rules and algorithms.	What level of signal changes from the outcome of interest would be considered significant and of clinical relevance?
Action (lessons 3 and 10): The *action* step describes the pre-specified interventions (actions) to be triggered in case of digital biomarker abnormalities by healthcare professionals or individuals.	What are meaningful interventions in case of significant digital biomarker deviations? Who is responsible for reacting to these deviations and in what timeframe?

### Feedback loops in the DACIA framework (orange box, informed by lessons 4, 7, 8, and 10)

During BarKA-MS, we regularly collected user feedback on the study and device acceptability in free-text fields. User studies were also conducted to identify healthcare professionals’ needs for data visualizations and considerations for appropriate data interpretation. This feedback was useful for study improvements. Therefore, since critical aspects for the study’s success may only surface during study conduct (e.g., through interim analyses or user feedback), we recommend that wearable sensor studies be adaptable to such feedback and evolving data requirements. This is visualized by the orange box in Fig. 2.

Regularly engaging participants through user feedback, e.g., as part of a weekly survey or after a data collection task has been completed, may also be beneficial for overall study compliance. In response to the feedback, researchers can promptly respond and provide motivational or technical support. The involved researchers can also keep support logs to record technical and non-technical issues that require further communication with participants. Considering participant burden, researchers should also assess the usefulness of individual data items during data collection, discarding those irrelevant to the study’s goals to reduce unnecessary burden. Researchers can also reduce burden by collecting data less frequently or re-using existing information, for example through linkage with clinical data.

Regular communication with study participants and healthcare professionals may also be useful for the interpretation of detected digital biomarker signals. Studies can explore implementing automated feedback loops to share deviating digital biomarker signals with study participants and healthcare professionals, gathering valuable data for process improvement or supervised machine learning models. These models should be critically assessed to ensure algorithmic fairness based on a diverse study population, to ensure that they are externally valid in other clinical settings and do not exclude underrepresented groups. Reviewing model results and predictions directly with involved stakeholders and diverse patient groups can help identify potential issues. Importantly, algorithms and digital biomarkers should also undergo external validation with independent patient populations before use in healthcare and clinical practice.

## Discussion

Our paper provides key lessons learned from the BarKA-MS study program for the use of wearable sensor data for digital biomarker development. Based on these, we propose the DACIA framework, which aims to guide and inform future research and support teaching curricula on digital health interventions. The framework is easily applicable to studies across various chronic conditions, in both observational as well as interventional study designs.

### The DACIA framework in the context of current guidance

In light of current guidelines, the DACIA framework provides interdisciplinary guidance on how to use wearable sensor data for digital biomarker development. Our work can be seen as complementary to other frameworks. The *Framework for Meaningful Measurement* by Manta et al.^52^, for example, provides a sequential list of data collection-related considerations to evaluate the meaningfulness of sensor signals. The *Digital Biomarker Discovery Pipeline* from Bent et al.^53^, goes a step further and focuses more specifically on aligning study goals with the collected data and different types of analyses. Guidance from Coravos et al.^9^ rather focuses on the variability in types of sensor technologies, digital biomarkers and their clinical relevance. Combined with high-level guidance from the FDA^15^ and Digital Medicine Society^16,17^, the DACIA framework provides a more comprehensive approach for planning and conducting research with wearable sensors to develop digital biomarkers that places focus on involving relevant stakeholders in each key step of DACIA in an iterative manner. This is especially of relevance in the *action* construct of the framework, going beyond digital biomarker development guidelines into meaningfully applying and assessing them along with relevant stakeholders in clinical practice. Furthermore, the DACIA framework places a more participant-centric approach that focuses on reducing their burden through support and continuous feedback. Overall, the DACIA framework complements existing guidance by focusing on participant needs as a crucial factor for study success, making it relevant for both short and long-duration studies.

### Implication for future studies

The DACIA framework fills an important gap by placing a stronger focus on the interdisciplinary and iterative planning, analysis and interpretation of wearable sensor data, to enhance the clinical relevance of future research in wearable sensor-based digital biomarker development. In particular, DACIA helps to assign the relevant responsibilities and clarify data requirements for assessing study outcomes and measurement contexts. It also underlines the importance of necessary measurement frequency to support relevant actions, such as by collecting user feedback and adapting the delivery of the study tasks based on this feedback in real-time, or regularly communicating with stakeholders to interpret and react to detected digital biomarker signals. While initially designed for the development of digital biomarkers from wearable sensors that measure physical activity, the DACIA framework can be applied to explore digital biomarkers using various devices or signal measurements, including for digital health interventions focused on behavior change.

An important consideration when implementing the DACIA framework in research studies is its applicability to larger study samples. BarKA-MS included 45 participants who received consistent support from the clinical staff and researchers to ensure completion of both the in-person and remote study components. The combination of a smaller sample size and the continuous support enabled higher personalization. However, we recognize that such approaches may not be directly applicable to larger studies or studies with limited resources. In the orange feedback loop of the DACIA framework, we propose approaches to streamline and automate certain study steps to reduce reliance on clinical staff and researchers. We also recommend referring to additional guidance documents^9,15–17,52,53^ and implementation science theories, such as the normalization process theory^54^, to further inform design actions that align smoothly with healthcare workflows, meet stakeholder needs, and utilize available resources efficiently.

### Strengths and limitations

This paper presents some limitations. The ten lessons are primarily derived from a single study program, which includes four published outcome analyses and a subsequent follow-up study, resulting in a relatively constrained experience base from a limited range of devices and data collection methods relevant to BarKA-MS. Moreover, the participant pool in BarKA-MS is limited to individuals with more advanced stages of MS, potentially limiting the generalizability of the findings to those living with other chronic diseases.

It is also important to note that the individual steps of the DACIA framework may not hold the same significance for certain applications and studies, particularly those that do not involve interventions. While we believe the DACIA framework adequately addresses important study design and conduct decisions relevant for digital biomarker development, we cannot rule out the possibility that certain studies may demand additional considerations beyond the scope of the framework. Therefore, further refinements and real-world testing are advisable.

Nevertheless, the DACIA framework builds on substantial research, data from wearable sensors and valid survey instruments, practical experience in conducting various digital health studies that use sensor measurements from wearables, and teaching experience with medical students. As such, we consider the framework to be well-grounded and reflective of real-world challenges in such studies, which can be informative for future research and teaching.

Overall, this paper outlines a set of important lessons learned for transforming wearable sensor data to digital biomarkers. The DACIA framework was developed as a crosscut between the lessons learned, which were summarized into five key steps of digital biomarker development and adapted based on student feedback. It highlights important elements to be considered when using wearable sensor data as digital biomarkers and provides practical guidance for future research and teaching. Our findings are applicable beyond MS and aim to inform any related digital health study for chronic disease management. As the popularity and use of wearables continuous to grow, our work provides an important first step towards the systematic and transparent development of meaningful digital biomarkers.

### Supplementary information

Supplemental material

## Data Availability

The data that support the findings of this study are available from the corresponding author upon reasonable request.
