# Supplementary material for: From wearable sensor data to digital biomarker development: ten lessons learned and a framework proposal
Source: NPJ Digit Med. 2024 Jun 18;7:161. doi: 10.1038/s41746-024-01151-3 (PMC11189504; doi:10.1038/s41746-024-01151-3)
Supplement: Supplementary file 1 — Supplemental material [file 41746_2024_1151_MOESM1_ESM.docx]

**Supplementary Table 1.**Study participants’ characteristics

| Characteristics | | | Study participants (n=45) |
| --- | --- | --- | --- |
| **Baseline demographics** | | | |
|  | **Sex, n (%)** | | |
|  |  | Female | 29 (64) |
|  |  | Male | 16 (36) |
|  | Age (y), median (IQR) | | 46 (40-51) |
|  | **Nationality, n (%)** | | |
|  |  | Swiss | 34 (76) |
|  |  | German | 6 (13) |
|  |  | Italian | 2 (4) |
|  |  | Other | 3 (7) |
|  | **Marital status, n (%)** | | |
|  |  | Single | 12 (27) |
|  |  | Married | 23 (51) |
|  |  | Separated | 1 (2) |
|  |  | Divorced | 7 (16) |
|  |  | Widowed | 2 (4) |
|  | **Education, n (%)** | | |
|  |  | Mandatory school not completed (or up to and including grade 7) | 2 (4) |
|  |  | Apprenticeship or secondary education completed (ie, *matura* schools or intermediate diploma schools) | 25 (56) |
|  |  | Higher professional education, universities of applied sciences, or university completed | 18 (40) |
|  | **Employment status, n (%)** | | |
|  |  | Working full time | 5 (11) |
|  |  | Working >50% but <100% | 5 (11) |
|  |  | Working ≤50% | 17 (38) |
|  |  | Not working | 18 (40) |
| **Baseline health information** | | | |
|  | **Multiple sclerosis type, n (%)** | | |
|  |  | Relapsing-remitting multiple sclerosis | 18 (40) |
|  |  | Primary-progressive multiple sclerosis | 8 (18) |
|  |  | Secondary-progressive multiple sclerosis | 19 (42) |
|  | Multiple sclerosis duration (y), median (IQR) | | 11 (5-21) |
|  | Expanded Disability Status Scale score, median (IQR) | | 4.5 (3.5-6) |
|  | **Expanded Disability Status Scale score, n (%)** | | |
|  |  | 0-3.5 | 15 (33) |
|  |  | 4-5.5 | 18 (40) |
|  |  | ≥6 | 12 (27) |
|  | **Time since last relapse (y)** | | |
|  |  | Value, media (IQR) | 3 (1-5) |
|  |  | Missing information, n (%) | 8 (18) |
|  | BMI (kg/m^2^), median (IQR) | | 24 (21-28) |
|  | **BMI (kg/m^2^), n (%)** | | |
|  |  | <18.5 (underweight) | 5 (11) |
|  |  | 18.5-24.9 (healthy weight) | 22 (49) |
|  |  | 25.0-29.9 (overweight) | 10 (22) |
|  |  | ≥30.0 (obesity) | 8 (18) |
|  | **Comorbidities, n (%)** | | |
|  |  | None | 18 (40) |
|  |  | Hypertension | 5 (11) |
|  |  | Depression | 5 (11) |
|  |  | Skin diseases (eg, acne) | 4 (9) |
|  |  | Orthopedic diseases (eg, joint or back pain) | 4 (9) |
|  |  | Type 2 diabetes | 3 (7) |
|  |  | Migraine | 2 (4) |
|  |  | Hypothyroidism | 2 (4) |
|  |  | Other | 9 (20) |
|  | **Change in the amount of sport practiced after the multiple sclerosis diagnosis, n (%)** | | |
|  |  | Less | 27 (60) |
|  |  | Same amount | 2 (4) |
|  |  | More | 15 (33) |
|  |  | Missing information | 1 (2) |
|  | Time spent at the rehabilitation clinic (d), median (IQR) | | 22 (18-26) |
|  | Barriers to Health Promoting Activities for Disabled Persons scale score at analysis baseline (ie, at the end of the rehabilitation stay; range 18-72; the higher the score, the more barriers to physical activity), median (IQR) | | 20 (19-21) |

**Supplementary Table 2.** Application of DACIA framework to BarKA-MS

| **Study name and goals** | | | **Application in BarKA-MS** | **Rationale** |
| --- | --- | --- | --- | --- |
|  |  |  | Assess the feasibility of sensor measurements to help people living with MS (PwMS) manage their symptoms through physical activity | |
| **D** | **Data** (Collection of sensor measurements and validation data) | Sensor measurements | **Data collection tools**: Fitbit Inspire HR, Actigraph GTX **Collected measures** (*level of granularity*): heart rate (*1-minute and 15-minute*), step count (*1-minute and 1-hour*), sleep quality (*1-minute*), physical activity intensity (*1-minute and 1-hour*), sedentary time (*1-minute and 1-hour*) and energy expenditure (*1-minute and 1-hour*) | Select the relevant device measurement parameter among that best matches the study outcomes of interest |
|  |  | Validation data | **Data collection tools**: survey instruments, in-person assessments **Collected measures**: Perceived Barriers to Physical Activity, 12-item MS Walking Scale-12, EQ-5D-5L, PHQ-8, GSE, FSMC, Pain assessment, 6 Minute Walk Test (6MWT), Meter Walk Test (10mWT), Timed Up and Go (TUG) | Select additional measurement instruments such as validated patient-reported outcomes.  The additional measurements can, for example be used for validation, analysis stratification, or cover additional physical and mental well-being dimensions. |
| **A** | **Aggregation** (Extraction and pre-processing of relevant features, aggregration and transformation according to most useful temporal granularity) | | **Data preprocessing**: removal of first wear day from analyses due to varying times when participants received the devices; calculate *valid wear time* for all participants (i.e., to consider days in analysis where participants wore the device for at least of 80% of the day)  **Data aggregation**: aggregate day- and week-level data on physical activity, including daily step counts and minutes in physical activity  **Data transformation**: create individual and cohort-level average values for all measures to calculate internal benchmarks | Define the most appropriate aggregation level:  seconds, minutes, hours, days, weeks, months per candidate biomarker |
| **C** | **Contextualization** (Combining processed sensor measurements with relevant contextual information) | | **Define benchmarks**: internal benchmarks (i.e., create cut-off based on certain patterns in data occurring more or less frequently than average); external benchmarks (e.g., categorized physical activity intensity calculated from the activity sensor) **Define sensor data for contextualization**: part of day (morning, afternoon, evening, night), day of the week, weather conditions **Define validation data for contextualization**: free-text responses, relevant measures from survey instruments **Define analyses**: manual linkage of sensor measurements with metadata; multi-level modeling with relevant covariates to exclude non-relevant explainable longitudinal variation | Define other data that will aid interpretation of digital biomarker patterns |
| **I** | **Interpretation** Denoising of time series, identification of relevant, unexplained signal deviations from internal or external norm | | **Identify sources of noise**: diurnal or weekly cycles, season, weather  **Identify relevant influences**: use survey instruments and in-person assessment data to validate whether observed anomalies or deviations in data to identify the underlying reasons (e.g., high levels of fatigue or pain in a certain day/week)  **Identify norm values**: identify within-person fluctuations and between-person fluctuations, and assess whether fluctuations are normal or benchmarks for digital biomarkers of interest (with help from literature-based normal ranges)  **Define digital biomarkers**: use internal benchmarks derived from between-person fluctuations to derive study-relevant digital biomarkers | Formulate literature- and experiance based hypotheses of possible contextual or personal influences on digital biomarkers of interest.  If possible, define literature-based normal ranges or benchmarks for digital biomarkers of interest |
| **A** | **Action** (Trigger appropriate, pre-defined actions to positively influence meaningful health aspect) | | **For healthcare professionals**: Use data to support PwMS to engage in regular physical activity at home; adapt rehabilitation program based on individual needs (e.g., if observe that individual measurement falls outside the norm of rest of cohort); possible development of aftercare program using the methods and results from the study  **For individuals**: Understand own motivators and barriers to engage in physical activity to better adhere to physical activity routines in the future; self-monitoring; monitoring of own progress; motivation to do more physical activity | Describe how the digital health intervention should be adapted if digital biomarkers of interest fall outside the norm or do not reach desired benchmarks. |
